# Novel alleles gained during the Beringian isolation period

**DOI:** 10.1038/s41598-022-08212-1

**Published:** 2022-03-11

**Authors:** Sara D. Niedbalski, Jeffrey C. Long

**Affiliations:** 1 Human Evolutionary Genetics Unit, UMR 2000, CNRS, Institut Pasteur, Paris, France; 2 Department of Anthropology, University of New Mexico, Albuquerque, NM 87131 USA

**Keywords:** Computational biology and bioinformatics, Evolution, Genetics

## Abstract

During the Last Glacial Maximum, a small band of Siberians entered the Beringian corridor, where they persisted, isolated from gene flow, for several thousand years before expansion into the Americas. The ecological features of the Beringian environment, coupled with an extended period of isolation at small population size, would have provided evolutionary opportunity for novel genetic variation to arise as both rare standing variants and new mutations were driven to high frequency through both neutral and directed processes. Here we perform a full genome investigation of Native American populations in the Thousand Genomes Project Phase 3 to identify unique high frequency alleles that can be dated to an origin in Beringia. Our analyses demonstrate that descendant populations of Native Americans harbor 20,424 such variants, which is on a scale comparable only to Africa and the Out of Africa bottleneck. This is consistent with simulations of a serial founder effects model. Tests for selection reveal that some of these Beringian variants were likely driven to high frequency by adaptive processes, and bioinformatic analyses suggest possible phenotypic pathways that were under selection during the Beringian Isolation period. Specifically, pathways related to cardiac processes and melanocyte function appear to be enriched for selected Beringian variants.

## Introduction

**Figure 1:**
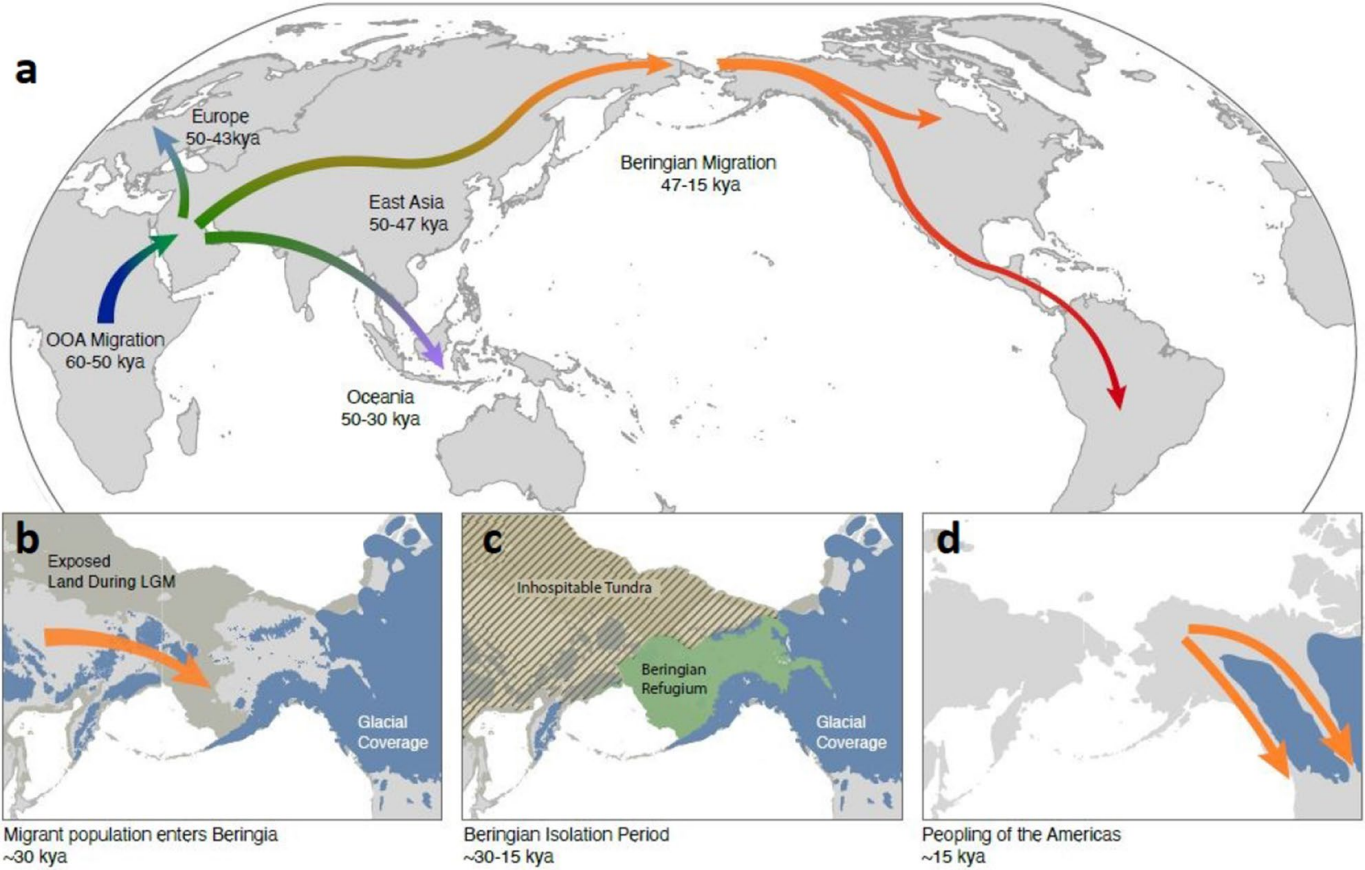
Global dispersal of humans recently inferred from genomic data^11–13^(**a**). Additional details specific to the Beringian Migration are given in panels (**b–d**). A migrant population entered the Beringian corridor by  30kya, during the LGM^10,14^(**b**). The Laurentide and Cordilleran Ice Sheets blocked entry into the American continents, while an inhospitable mesic tundra developed in Eastern Siberia, preventing backward movement^15,16^. The migrant population was thus isolated for upwards of 15 ky in a Beringian refugium (**c**) until glacial retreat exposed coastal and interior routes into North America (**d**). To create panel (**a**), we drew the outline of continents using the R package maptools version 1.1-2 (https://cran.r-project.org/web/packages/maptools/index.html) and then added the labels and paths of migrations as overlays in Adobe Illustrator. We generated panels (**b–d**) by adding shading and overlays to portions enlarged from panel (**a**).

The Beringian migration marks one of the most striking events in modern human history. Genetic and archaeological data confirm that a small population consisting of a few thousand people entered the Beringian corridor from Siberia at the advent of the Last Glacial Maximum (LGM), approximately 30 thousand years ago (kya)^1–5^. The Beringian ecology provided a refuge for this migrant population as the LGM intensified^6^. Plant macro-fossils and fossil pollen from Beringia suggest that it was a productive dry grassland ecosystem^7^ inhabited by a variety of large mammals^8^. However, North American glacial coverage and inhospitable Siberian environments during the LGM effectively sealed off the migrant population in the Beringian refugium, preventing either forward or backward movement until approximately 15kya when the surrounding glaciers receded, opening up both coastal and interior corridors of entry into the North American continent^2,4,9,10^ (Fig. 1).

The demographic and paleo-ecological features of the Beringian experience have been well characterized^2,7,17,18^. However, the genomic impacts of the Beringian experience are still being discovered. Several factors suggest that there was a great deal of opportunity for genetic evolution in the Beringian population. Importantly, the population originated from a small group of founders and maintained a small size for millennia^4,19–21^. The combination of a founder effect and prolonged bottleneck would have greatly enhanced genetic drift^22^. It is well-known that genetic drift will reduce variation and provide a descendant population with only a subset of the variation that was present in their ancestors^23^. The current literature documents such a reduction in variation, and the subset pattern, in Native Americans in comparison to Eurasians and Africans^24^. However, in addition to the loss of alleles, genetic drift can elevate the frequencies of rare alleles and new mutations^25–27^. This will occur to a much lesser extent than the loss of standing variation. Nonetheless, full genome analyses make it possible to observe instances of such **‘allele gains’**. The gain of novel variation tracing back to the Beringian occupation has been less studied than the loss of variation. Allele gains are the major focus of this paper.

We expect that most alleles gained through founder effects and bottlenecks will be outside of gene coding and regulatory sequences, and therefore selectively neutral. However, it is also possible that some of the alleles gained through enhanced genetic drift will affect the expression of phenotypes. A portion of the alleles gained may have health consequences. In addition to genetic drift, positive natural selection by Beringian environmental conditions may have produced some allele gains. Indeed, a strong signature of positive selection has been found in several variants contained in the fatty acid desaturase (*FADS*) gene cluster, potentially modulating a unique lipid profile in response to a protein-rich diet^28^. Similarly, Hlusko and colleagues^29^ have argued that an amino acid substitution in the ectodysplasin A receptor (*EDAR*) may have evolved in response to vitamin D deficiency created by the low UV at high latitude. A survey of the genome may reveal more locations of adaptive changes.

The Beringian people are deep ancestors of all contemporary Native Americans^10^. They are also ancestors to populations that were formed in post-colonial times by admixture between Native Americans and people with ancestors in Europe and/or Africa^30,31^. As such, we can expect that the genetic changes acquired by either genetic drift or natural selection in Beringia will be wide-spread throughout populations with Indigenous American ancestry, but absent in all other people of the world. This provides us with a way to identify the allele gains that were made during the Beringia Isolation Period.

Here, we perform a full genome investigation to identify allele gains that were made by Native American ancestors during the Beringian Isolation Period and inherited by contemporary populations. Then, we perform a bioinformatic analysis to investigate possible functional consequences of these uniquely American alleles.

## Results

### Group specific polymorphisms

We found alleles gained during the Beringian Isolation Period by applying the concept of Group Specific Polymorphism (GSP). A GSP is a common allele in one group of people that is absent or nearly absent in all other groups. After a founder effect, GSPs will be present in both the ancestral and descendant populations. Ancestral and descendant populations can be distinguished after a founder effect by the mix of ancestral and derived alleles. GSPs in the ancestral population will be composed of a mix of ancestral and derived alleles. By contrast, GSPs in the descendant population will be almost entirely derived alleles that were gained from the founder effect. We analyzed whole autosome DNA sequences from the Thousand Genomes Project Phase 3 (TGPP3) sample to identify Group Specific Polymorphisms (GSP).

**Figure 2:**
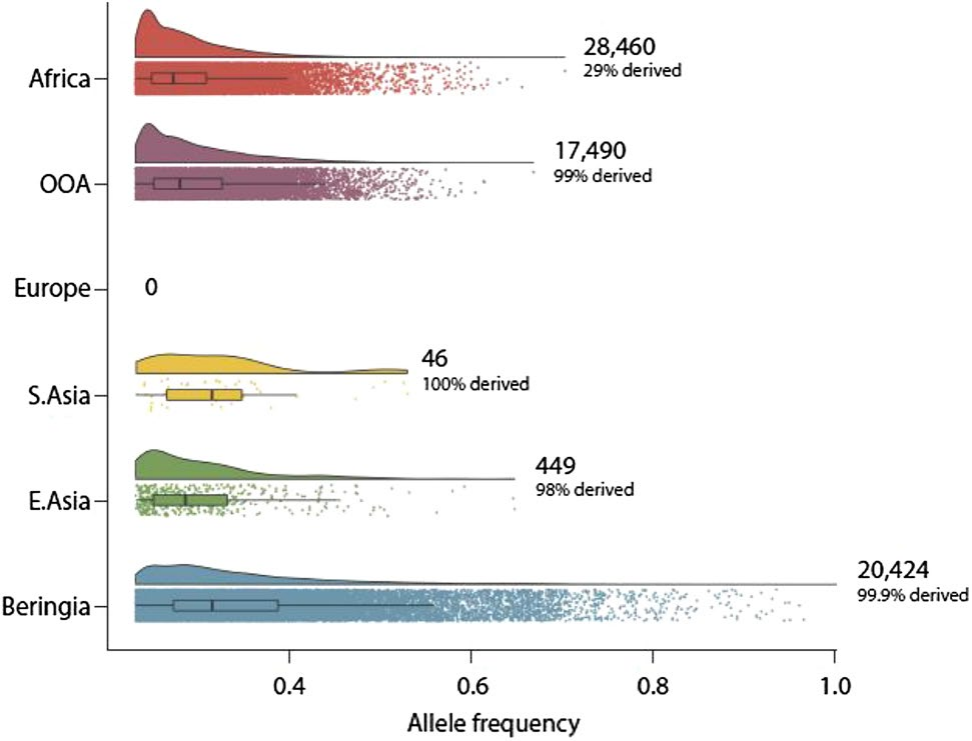
Global distribution of GSPs in the Thousand Genomes Project. Plots depict the density of GSPs for four continental populations, and two groups descended from founder effects and population bottlenecks. OOA designates the descendants of the out-of-Africa migration, and Beringia designates the descendants of the Beringian Founder Population. For each population group, the vertical axis displays the smoothed density (upper) and scatter (lower) of GSPs that have an allele frequency equal to the value given on the horizontal axis.

Figure 2 presents group specific polymorphisms for six broad groups of populations. The descendants of the Beringian migration harbor 20,424 GSPs. We found Beringian GSPs by examining the DNA sequences of people with mixed ancestry living in the Americas after controlling for European and African admixture. Only two other geographic divisions of our species showed comparable numbers of GSPs. A total of 28,460 GSPs were found in African people, represented by 5 populations living in Sub-Saharan-Africa. A total of 17,490 GSPs were found in descendants of the out-of-Africa (OOA) migration, represented by a total of 15 populations living in Europe, South Asia, and East Asia. Surprisingly few GSPs were found in Europeans (0, GSPs), South Asians (46, GSPs), and East Asians (449, GSPs). The GSPs in African populations were composed of a mix of ancestral (71%) and derived alleles (29%). The vast majority of GSPs in out-of-Africa descendants and Beringian descendants were derived alleles (99% and 99.9%, respectively). With only two exceptions, the group specific polymorphisms have not reached fixation. Both exceptions occur in the descendants of the Beringian migrants.

### Simulation results

We used coalescent simulations to verify that the observed pattern of derived GSP alleles is consistent with the reduction in heterozygosity that was seen in short tandem repeat and single nucleotide polymorphism data sets that support the serial founder effects model for genetic diversity in contemporary human populations^24,32^.

**Figure 3:**
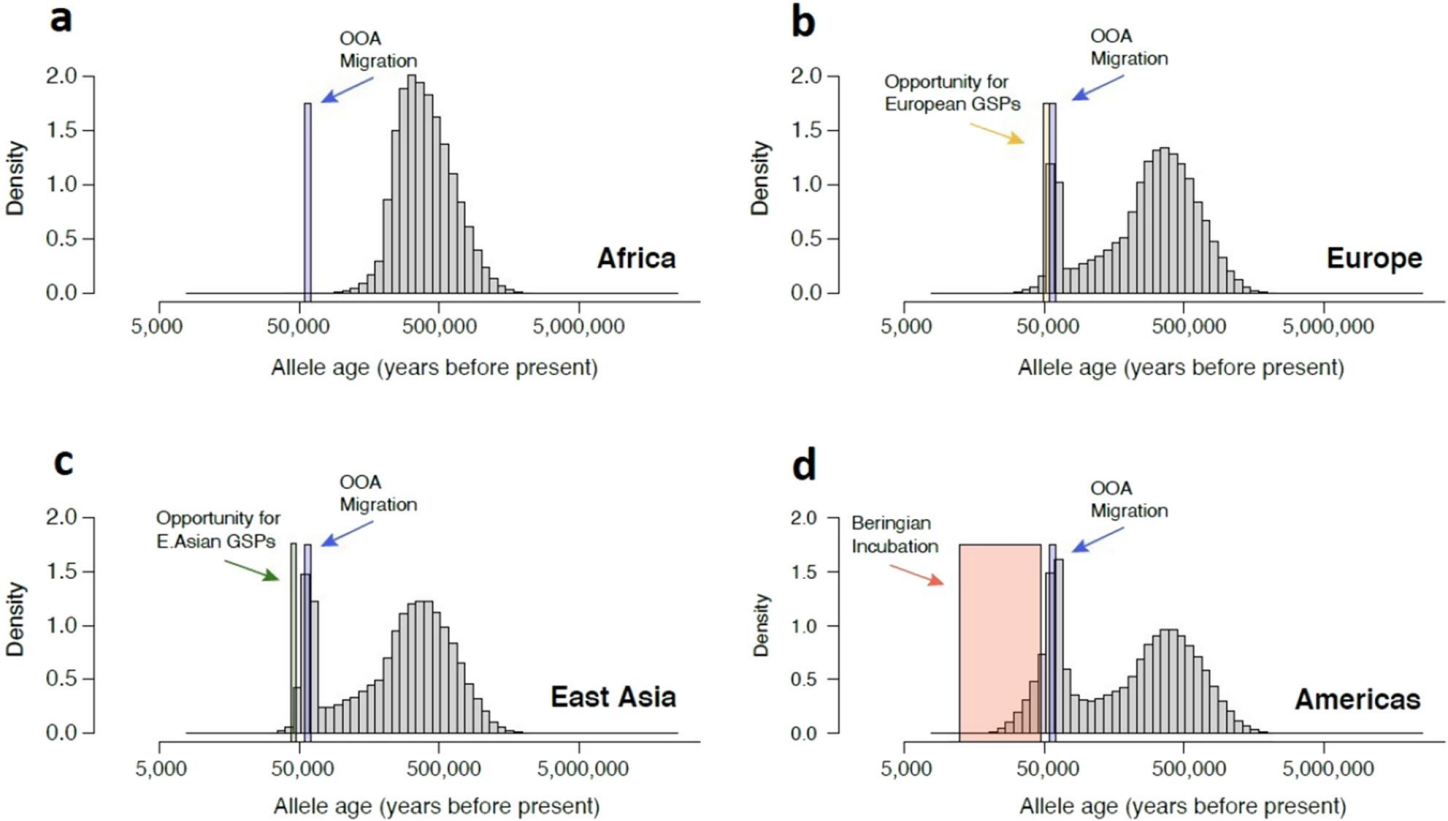
Simulated distribution of derived allele ages. Panels show the age distribution of high frequency ($p \ge 0.30$) derived alleles for Africans (**a**), Europeans (**b**), East Asians (**c**), and Native Americans (**d**). The demographic parameters of the coalescent simulations were calibrated by fitting a serial founder effects model anchored by archaeological dates to short tandem repeat data (details in text). Windows of opportunity for the emergence of private alleles during each migration/expansion are highlighted in colored boxes. Windows span the periods of 65–55kya for the out-of-Africa migration (blue), 55–49kya for expansion across Europe (green), 55–45ky for the expansion into East Asia (gold), and 47–20kya for the Beringian Isolation (orange).

Figure 3 shows the probability density for the age of a derived allele with frequency $p \ge 0.3$ for each of four geographic regions. The allele age distribution for an African population illustrates the great antiquity of human polymorphism Fig. 3a. The blue vertical bar marks the time window for the out-of-Africa migration 55,000–60,000 years ago. The chance that a derived allele in this frequency range in Africa will be older than this time window is nearly 100%. As such, common derived alleles in contemporary Africans were likely common alleles at the time of the out-of-African migration. They would have been present in the gene pool that gave rise to the out-of-Africa migrant population. Their absence in a contemporary non-Africans can be explained by genetically drifting out of the original out-of-African migrants and their immediate descendants.

Panels 3b-d show the probability density of the age of a derived allele with frequency $p \ge 0.3$ for a population in Europe, East Asia, and the Americas, respectively. The blue bar in each of these graphs again shows the time window of the out-of-Africa migration. The allele age probability spikes in this interval because founder effects such as the one that occurred with the OOA migration allow a few new mutations to rise to high frequency. The three non-African populations share this spike because they are all descendants of the original OOA migrants. This spike corresponds to the large number of non-African GSPs. The green bar in Fig. 3b marks the time window bracketed by the OOA migration and the diversification of European populations. There is very little area under the curve during this time window, and consequently there was a very small chance that an allele would fulfill the criteria required for a European GSP. The situation in a simulated East Asian population illustrates the same phenomenon. The gold bar in Fig. 3c brackets the time window between the OOA migration and the entry into East Asia and the diversification of East Asian populations. There is very little area under the curve during this time window. The orange bar in Fig. 3d brackets the time window between the separation of Native American ancestors and the ancestors of East Asians and the entry of Native American ancestors into the Americas. This is the Beringian Isolation Period. There is considerable area under the curve in this time window. Accordingly, this result predicts a substantial number of Native American GSPs would arise during the Beringian Isolation Period.

### Functional annotation of Beringian GSPs

**Figure 4:**
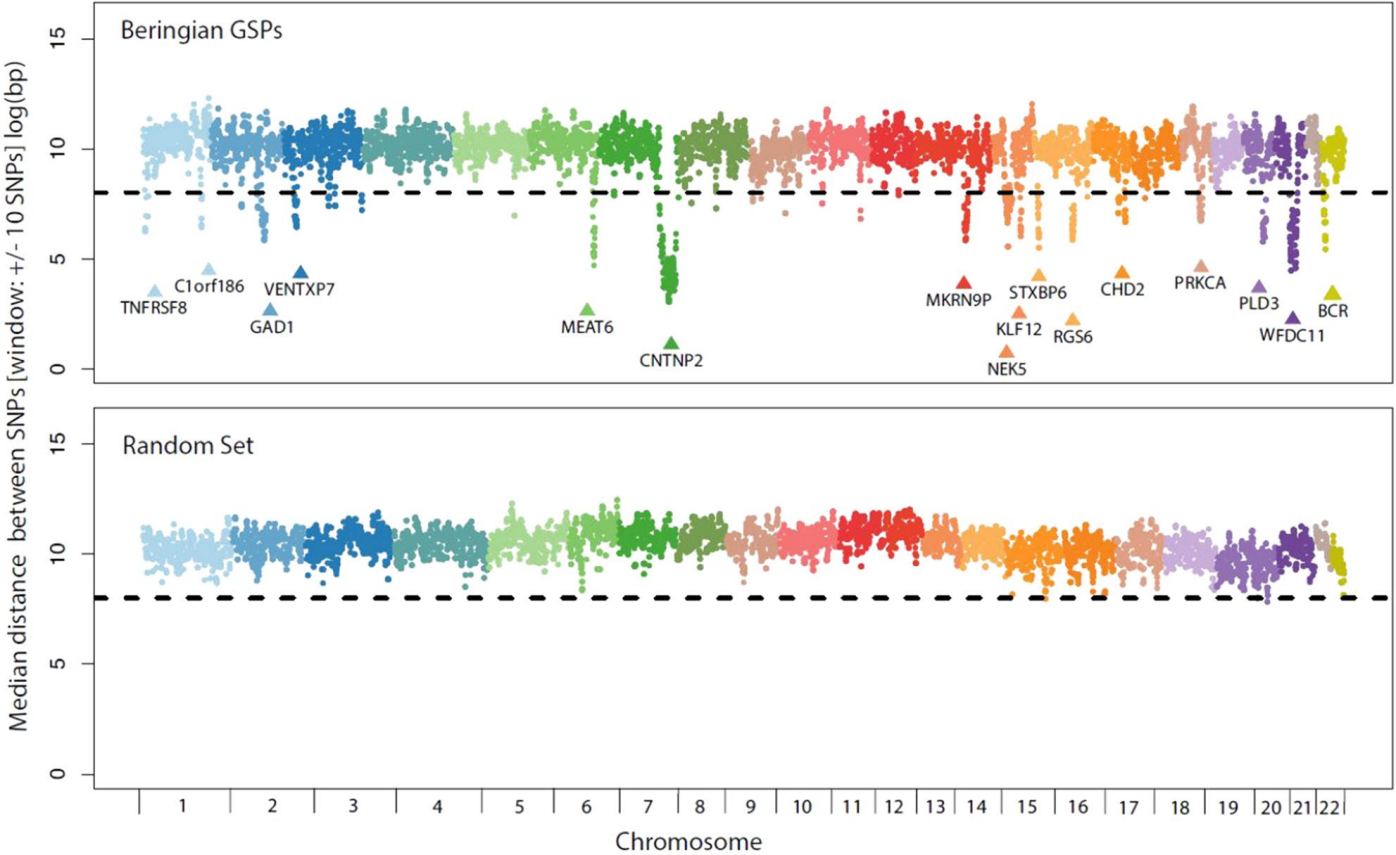
Positional enrichment within the set of Beringian GSPs. Each SNP in the set of Beringian GSPs and a matched random sample of Native American SNPs that did not meet our GSP criteria are plotted as their distance to the nearest neighbor SNP in the set. The dotted line is positioned at the 99.9th percentile. At least 16 notable regions of the genome contain groups of Beringian variants that are more closely clustered that would be expected by chance. The gene symbols for each cluster are labeled in the figure.

Figure 4 (upper) shows the spatial distribution of Beringian GSPs along chromosomes as the median distance between a GSP to it’s nearest neighbor in a sliding window of +/− 10 SNPs. Sixteen distinct clusters of densely packed Beringian SNPs emerge. The three largest clusters include 273 GSPs in the region of the Contactin Associated Protein 2 (*CNTNP2*) gene on chromosome seven, 69 variants in the region of the Makorin Ring Finger Protein (*MKRN9P*) gene on chromosome twelve, and 62 GSPs in the region of the Melanoma-Associated Transcript-6 (*MEAT6*) gene on chromosome six. Figure 4 (lower) shows the spatial distribution of the 20,424 matched polymorphisms along the chromosomes. Notice that the matched polymorphisms do not form clusters such as are apparent in the Beringian GSPs.

The Beringian GSPs located in genic versus inter-genic regions of the genome are presented on the first line of Table 1. The second line presents the same categorization of SNPs in the Random SNP Set. These data make it clear that Beringian GSPs are over-represented in the protein coding sequences relative to the Random SNP Set $\chi ^2 = 212.01, d.f. =2, p < 0.001$).

**Table 1:** Genomic distribution of GSPs.

SNP set	Protein coding	ncRNA	Inter-genic
Beringian GSP	9068	1206	10,090
Random set	7761	1225	11,642
	$\chi ^2 =212.01$, $\hbox {d.f.} = 2$, $\hbox {p} < 0.001$		

**Figure 5:**
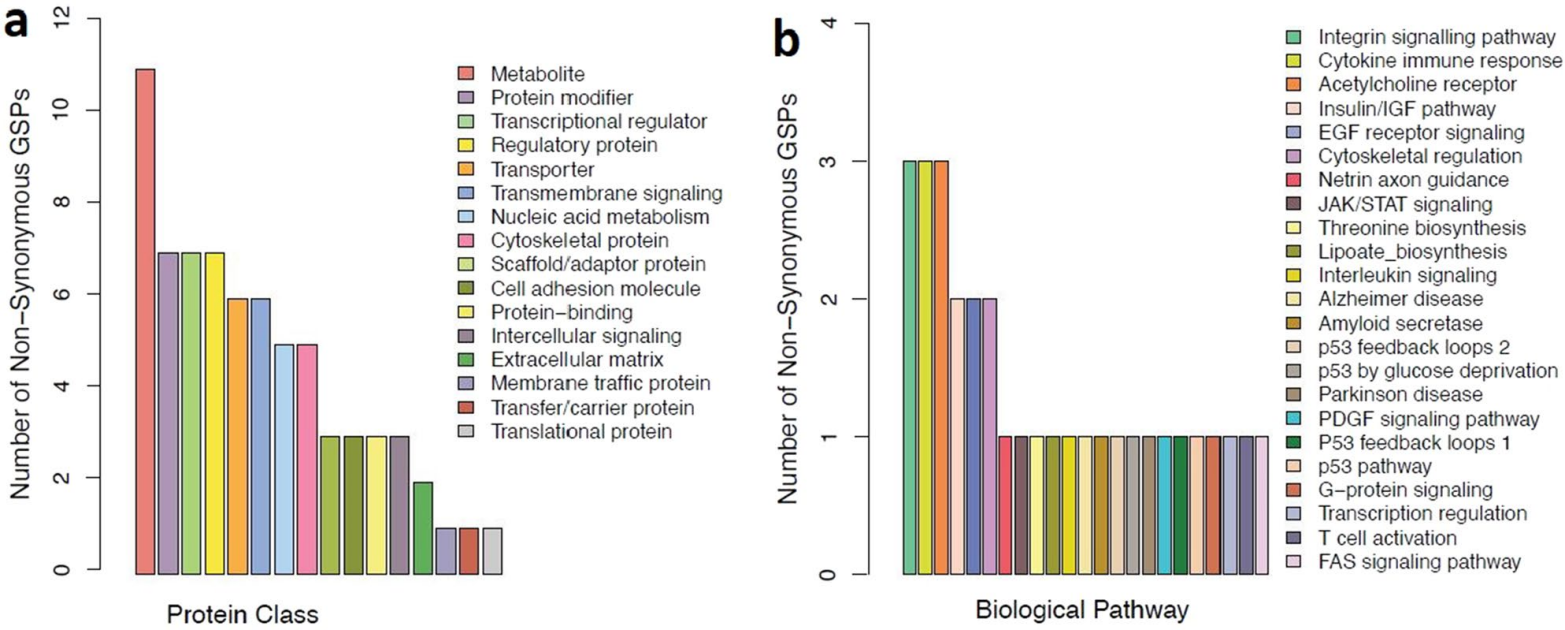
Panther annotations for non-synonymous GSPs. Panel (**a**) depicts the protein classes affected by non-synonymous variation unique to Beringia, and panel (**b**) annotates these protein coding changes according to which biological pathway they are implicated in.

Non-synonymous GSPs in protein coding sequences affected a number of different protein classes and biological pathways according to a Panther^33^ analysis (Fig. 5). Eleven genes that code for metabolite proteins contained non-synonymous GSPs (Fig. 5a). Additional categories containing non-synonymous GSPs include protein modifying enzymes, transcription regulators, and regulatory proteins. Amongst the biological pathways impacted by non-synonymous GSPs, the categories with the greatest number of genes include integrin signalling, cytokine-mediated immune response, and nicotinic acetylcholine receptors (Fig. 5b). Interestingly, four different biological categories associated with the p53 tumor suppression pathway were affected by non-synonymous GSPs. Supplementary Figure S1 compares these categories to a random set of non-synonymous GSPs.

### Evidence for natural selection

We have examined the ratio of non-synonymous to synonymous substitutions in the Beringian GSPs to detect the impact of the Beringian Isolation Period on the efficacy of purifying selection. Table 2 tabulates the percentages of Non-synonymous and Synonymous substitutions in the protein coding SNPs for the Beringian GSPs and Random SNP sets. In protein coding sequences, the proportion of non-synonymous relative to synonymous GSPs is $40.9\%/59.1\% = 0.69$. Natural selection against deleterious variation is evident because this rate is well-below unity, the expectation for selective neutrality. However, it is 3.14 times greater than the rate of non-synonymous relative to synonymous SNPs in the Random SNP set $18.0\%/82.0\% = 0.22$. This increase in non-synonymous SNPs may respresent a relaxation of selection against deleterious alleles. It is also noteworthy that given the high frequencies of these alleles, their functional consequences may affect many people.

**Table 2:** Non-synonymous and Synonymous Substitutions.

SNP set	Non-synonymous	Synonymous
Beringian GSP	3709	5359
Random set	1397	6364
	$\chi ^2 =1{,}036.6$, $\hbox {d.f.} = 1$, $\hbox {p} < 0.001$	

**Figure 6:**
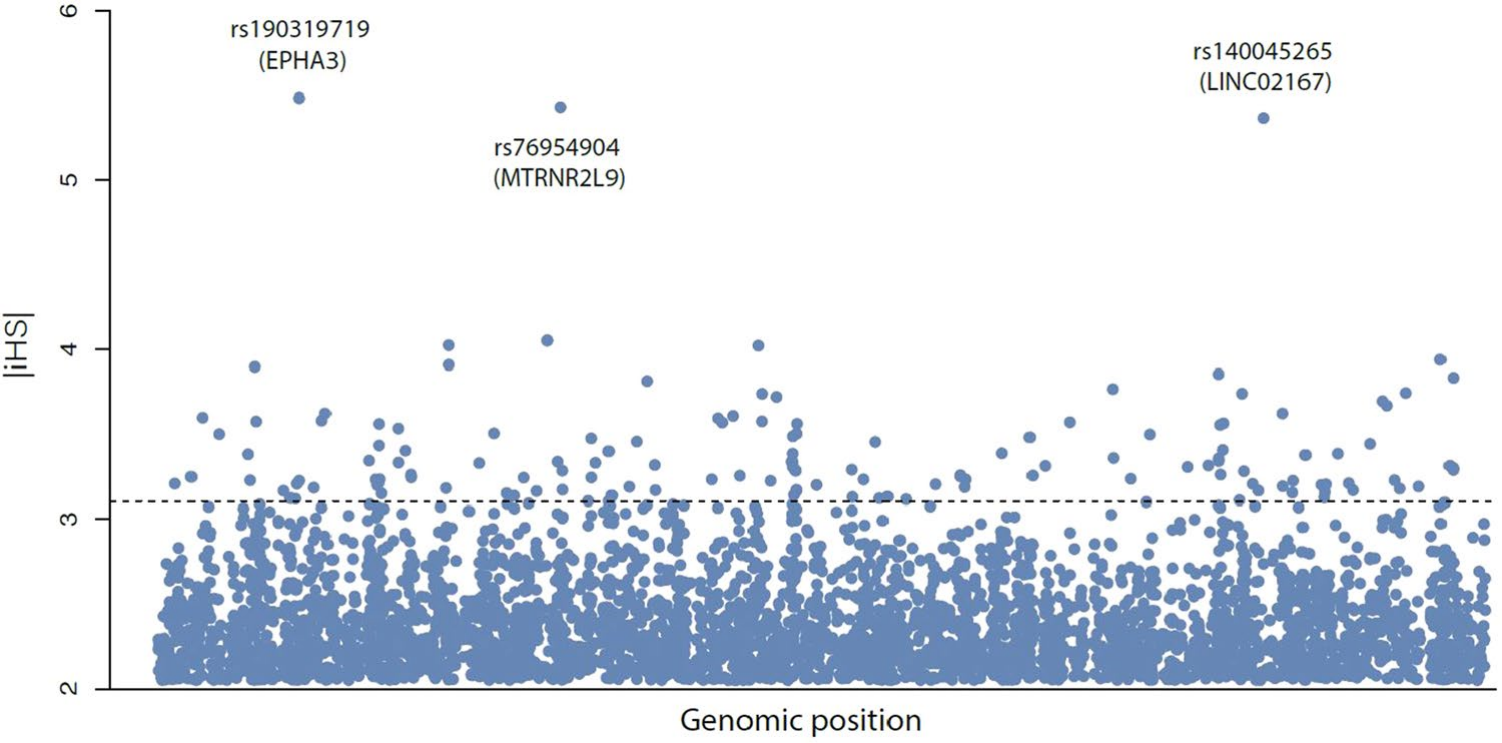
iHS scores for Beringian GSPs. The dashed line indicates the threshold for |*iHS*| scores within the top 5% for the entire genome 141 GSPs fall above this threshold, and three GSPs stand out as extreme outliers.

Extreme environments, such as high latitude, provide opportunities for environmental adaptation through positive selection. We computed integrated haplotype homozygosity scores (*iHS*) for the 20,424 SNPs in the GSP set to test the hypothesis that some Beringian GSPs are environmental adaptations. These tests yielded 2,820 candidate loci with ihs scores exceeding the generally accepted threshold |2.5|. Typically, in tests for selection where many loci pass a minimum significance threshold, only the outliers in the top 1-5% are considered. Here, the top 5% of significant *iHS* scores across the entire genome includes 141 Beringian GSPs (Fig. 6).

Three GSPs present extreme outliers with |*iHS*| values above 5: rs190319719 ($\hbox {iHS} = -5.48$) within the gene *EPHA3*, rs76954904 ($\hbox {iHS} = -5.43$) in *MTRNR2L9*, and rs140045265 ($\hbox {iHS} = -5.36$) in *LINC02167*. There is a sizeable gap between these outliers and the next greatest |*iHS*| (rs141503817, $\hbox {iHS} = -4.05$). We have also considered genes disproportionately affected by multiple SNPs under positive selection. Table 3 reports genes containing the most SNPs within the top 5% of iHS.

**Table 3:** Genes containing the greatest number of SNPs within the top 5% of iHS scores.

Gene	Chr.	Selected SNPs$^{1}$	Beringian SNPs$^{2}$	$|iHS|^{3}$	Putative function$^{4}$
TYRP1	9	7	66	3.30	Melanin production
PTPRD	9	5	42	3.37	Regulation of cell growth, differentiation, tumorigenesis
GOLGA6L4	15	3	10	3.52	Associated with BMI-adjusted waist circumference
RIPK4	21	3	3	3.47	Stratified epithelial development and keratinocyte differentiation
UGT2B7	4	3	8	3.40	Elimination of toxic xenobiotics and endogenous compounds

$^{1}$ Number of selected Beringian GSPs in the listed gene.

$^{2}$ Total number of Beringian GSPs in the listed gene.

$^{3}$ Averaged |*iHS*| scores for selected GSPs.

$^{4}$ Functional annotations compiled from GeneCards database^34^.

**Figure 7:**
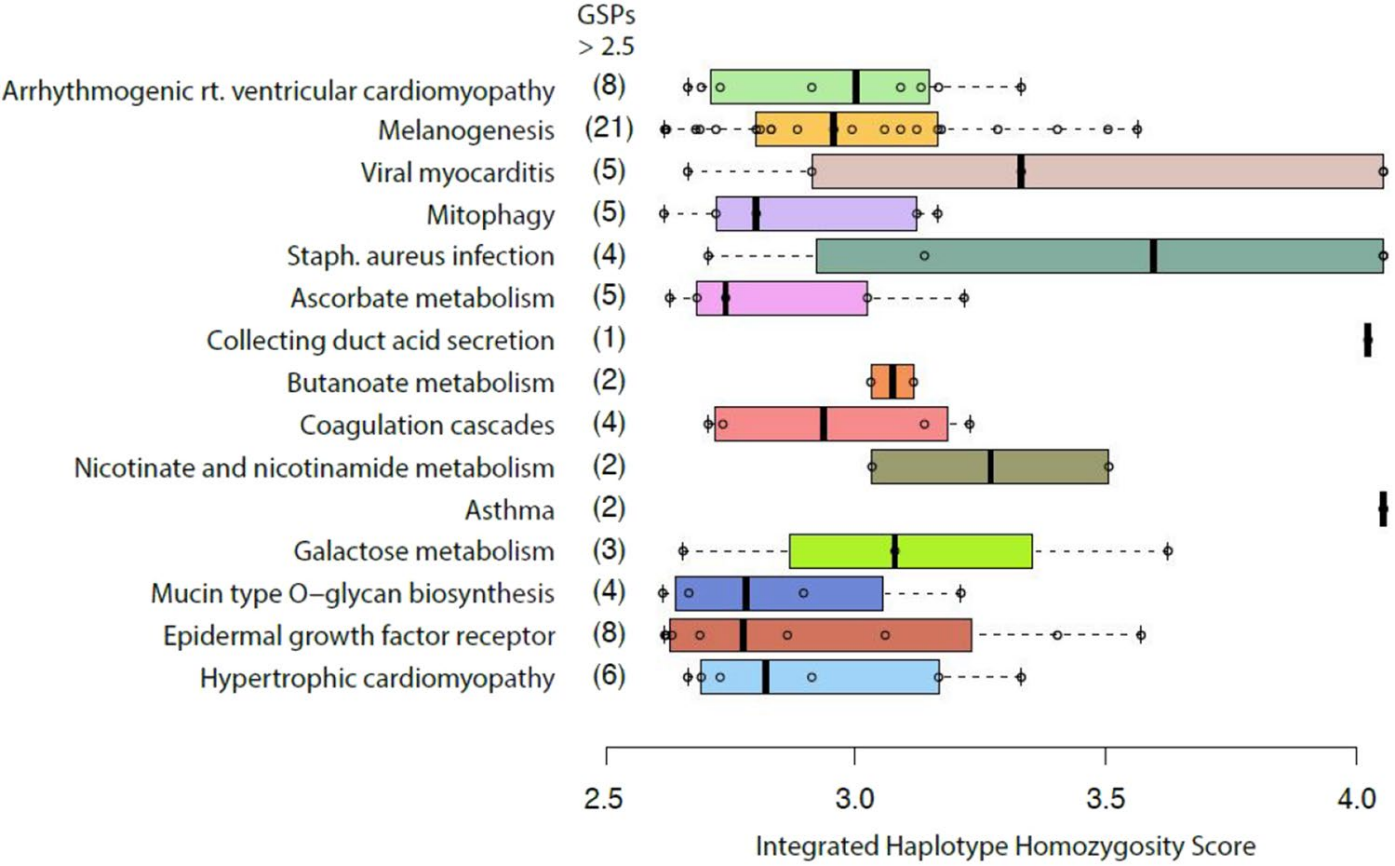
Top 15 KEGG pathways enriched for Beringian GSPs in the top 5% of selected *iHS* scores. Box and whisker plots are ordered (top to bottom) by ENRICHR Combined Scores, which provide a relative ranking of categories in order of their deviation from expected rank. Categories with higher enrichment scores are ranked much higher than expected by chance, while those with lower scores are closer to their expected rank. Box and whisker plots include all Beringian GSPs with |*iHS*| > 2.5 that fall in each category.

Figure 7 displays functional categories enriched for positively selected ($iHS > 2.5$) Beringian GSPs . By looking at multi-gene pathways impacted by positive selection, we can begin to get a sense of possible polygenic adaptation affecting complex traits. The KEGG pathway with the greatest combined enrichment score identified in our analysis is arrhythmogenic right ventricular cardiomyopathy (ARVC) with eight selected GSPs affecting genes related to this pathway. Within the top 15 categories, at least two other KEGG pathways suggest adaptive evolution in pathways related to cardiac function. Interestingly, the second most enriched category is melanogensis, with 21 selected GSPs across 6 genes related to the production of melanin in the skin, hair, and eyes.

## Discussion

The Beringian Isolation Period encompassed the last of a series of major founder effects that occurred during the peopling of the world. Heretofore, the principal evidence for these founder effects is a decline in heterozygosity in populations that is proportional to the geographic distance of a population from Sub-Saharan Africa^24^. However, reduction in heterozygosity is not the only consequence of founder effects and bottlenecks. These phenomena will occasionally elevate the frequencies of new mutations and rare alleles. Such ‘allelic gains’ from founder effects and bottlenecks have been less well-studied. We show that the Beringian founding population gained many unique alleles during its isolation, and that these alleles are shared widely among its contemporary descendants.

We found alleles gained during the Beringian Isolation Period by applying the concept of Group Specific Polymorphism (GSP). A GSP is a common allele in one group of people and absent or nearly absent in all other groups. As such, a GSP will be diagnostic of ancestry from that group, and conversely, group membership will be a reasonable indicator that an individual will carry the allele. After a founder effect, GSPs will be present in both the ancestral and descendant populations. However, the ancestral and descendant populations can be distinguished by the mix of ancestral and derived alleles. GSPs in the ancestral population will be composed of a mix of ancestral and derived alleles, whereas GSPs in the descendant population will be almost entirely derived alleles. Our analyses reveal GSPs in only three groups: Sub-Saharan Africans, Eurasians, and admixed Americans. Sub-Saharan African GSPs have the greatest number of GSPs (28,460). These alleles clearly identify Sub-Saharan Africans as the ancestral population as 71% percent are the ancestral state and 29% are the derived allele state. By contrast, the set of Eurasian GSPs is composed almost exclusively of derived variants (99%), and further document that despite some archaic admixture, the Eurasian gene pool was primarily established by the Out of Africa (OOA) migration. The American GSPs are composed of 99.9% derived alleles and show the Beringian founder effect. It was surprising at first glance that GSPs specific to European, South Asian, and East Asian populations are absent or rare. However, the results are consistent with our simulations of the serial founder effects model and are easily explained by the fact that Eurasia was settled in a narrow time window after the out of Africa migration.

One of the most striking features of Beringian GSP architecture is the distribution of GSPs throughout the 22 autosomes. Over 90% of the GSPs are distributed evenly with the spacing pattern approximating that seen in the set of 20,424 random SNPs. The evenness in the GSP distribution is punctuated by distinct clusters in 16 chromosomal regions (Fig. 4). There are genes associated with each cluster, but whether or not these GSPs influence the products or expression of these genes is an open question. We note that the random SNP set does not eliminate the possibility that micro-evolution in the Beringian Isolation Period would have caused clustering in common polymorphisms that are not GSPs. However, there are two important points in interpreting these features. First, Native American ancestry in the CLM, MXL, and PEL accounts for many of the non-GSP common polymorphisms in these populations. In this light, we would have expected to see clusters if they had formed, however the clusters might be somewhat attenuated because they are older . Second, the absence of similar clusters in the random SNP set suggests either that the out-of-Africa migration did not form such clusters, or else, the greater antiquity of that migration has allowed enough time for recombinations to randomize such clusters. This expectation follows from the fact that the major components of ancestry in the CLM, MXL, and PEL samples are Native American and European, and both of these groups of people descended from the Out-of-Africa migrants.

Two lines of evidence indicate that purifying selection has been relaxed in the alleles gained during the Beringian Isolation Period. First, the percentage of GSPs in coding sequence (44.4%) significantly exceeds the percentage of Random SNPs in coding sequence (38%). Second, in Beringian GSPs that do occur in coding sequence, the ratio of non-synonymous to synonymous nucleotide substitutions ($\omega = 0.69$), is substantially higher than the corresponding ratio in the Random SNP set ($\omega = 0.22$).

We have used the iHS statistic to identify a set GSPs that are candidates for positive selection. These comprise a small fraction of Beringian GSPs 141/20,424 = 0.0069). The relevant phenotypes that favored survival and reproduction of individuals cannot be directly inferred from the nucleotide sequence data alone. Therefore, we have used bioinformatic analyses to gain provisional insights into potential phenotypes influenced by these alleles. Our analyses were performed by parsing the candidate loci according to three criteria, individual SNPs with outlying iHS scores, specific genes that harbor a disproportionate number of putatively selected GSPs, and gene ontology classes enriched for putatively selected GSPs. In combination, these three lines of evidence suggest adaptations in the Beringian Isolation Period are related to cardiac function and melanogenesis.

The gene *EPHA3* which includes the GSP with the most extreme iHS score (rs190319719, $\hbox {iHS} = -5.48$) encodes a tyrosine kinase receptor that has been shown to important in cardiac cell migration and differentiation, and in regulating the formation of the atrioventricular canal and septum during development. Similarly, the Arrhythmogenic Ventricular Cardiomyopathy, Viral Myocarditis, and Hypertrophic Cardiomyopathy KEGG pathways are enriched with GSPs showing evidence of positive selection. Twenty-one GSPs showing positive selection appear in the melanogenesis pathway. *TYRP-1*, which contains 5 unique variants under positive selection, ranking highest amongst individual genes targeted by selection in Beringia. It is an intriguing possibility that selection on genes involved in melanocyte function could have favored depigmentation to increase biosynthesis of vitamin D^29,35^ in a low UV environment.

Altogether, the analyses we present in this paper emphasize the importance of the Beringian Isolation Period for generating unique genomic variation that distinguish Native Americans from other continental groups. The magnitude of this effect relative to the effect of the Out-of-Africa migration underscores the importance of major bottleneck events for the evolution of unique group-specific allele gains in continental populations. Further, the evolutionary approach we demonstrate in this paper has a wealth of potential for insight into population differences in molecular phenotypes relevant for health and disease. Functional studies that link these variants to particular biological phenotypes stand to generate new insights into pathways underlying population disparities in health and disease and may uncover novel candidate genes that may one day serve as potential therapeutic targets.

## Methods

### Genomic data

We analyze whole autosomes from the Thousand Genomes Project Phase 3 (TGPP3) sample to identify SNPs originating in the ancient Beringians (Table S1)^36^. Briefly, the total TGPP3 data consist of 84.7 million single nucleotide polymorphisms (SNPs) determined from next-generation sequencing of 2,504 individuals. Each individual was sequenced for the whole genome using mean depth $= 7.4\times$ enhanced by sequencing targeted exomes at mean depth $= 65.7\times$. The TGPP3 sample includes populations from five geographic regions: Africa (five populations, total $\hbox {N}=504$), Europe (five populations, total $\hbox {N} = 503$), South Asia (5 populations, total $\hbox {N}=489$), East Asia (5 populations, total $\hbox {N}=504$) and the Americas (6 populations, total $\hbox {N} = 504$). We use data from all TGPP3 populations residing outside of the Americas and three populations with substantial Native American ancestry that currently reside in the Americas.

These are ($\hbox {N}=64$) individuals with Mexican ancestry from Los Angeles, California (MXL), ($\hbox {N}=85$) individuals from Lima, Peru (PEL), and ($\hbox {N}= 94$) individuals from Medellin, Colombia (CLM). These three populations formed through admixture among Native Americans, European colonists, and African slaves during the colonial period beginning in the $15^{th}$ century. These three populations have substantial Indigenous American ancestry. Martin and colleagues report the degrees of Indigenous American ancestry for each population; Peruvian (77% ), Mexican American (47%), and Colombian (26%)^37^. Taken together, the Indigenous American proportions are equivalent to approximately 119 unadmixed genomes. The approach outlined below extracts information about genetic variants contributed by Indigenous American ancestors shared by all three populations.

### Group specific polymorphisms (GSPs)

**Figure 8:**
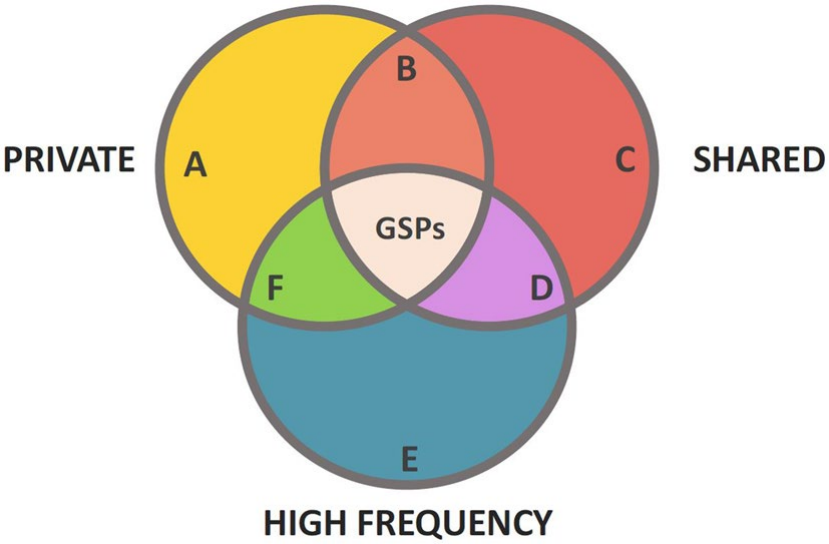
Group Specific Polymorphisms. We define Group Specific Polymorphisms (GSPs) as alleles that meet the following criteria: high frequency ($p \ge 0.30$), private to the group, and shared by all sub-populations within the group.

We define a group specific polymorphism (GSP) as an allele that is at high frequency within a group of populations, private to that group of populations, and shared by all populations within the group (Fig. 8). As such, a GSP will be diagnostic of ancestry from that group, and conversely, group membership will be a reasonable indicator that an individual will carry the allele. Operationally, we required a GSP to (1) be present in all populations belonging to the group for which it is defined, (2) to have an allele frequency greater than 30% in the focal group, and (3) to have an allele frequency less than 1% in all populations and outside the focal group. The 30% and 1% thresholds were pragmatic choices for this study, guided by broad patterns in the human species. By applying the Hardy-Weinberg principle, we see that the expected probability that a group member carries a GSP using the 30% and 1% criteria has an approximate minimum of $(0.3)^2 + 2(0.3)(07) = 0.51$, whereas the expected probability that a member of a different group carries the GSP has an approximate maximum of $2(0.01)(0.99) + (0.01)^2 = 0.02$. The actual probabilities will depend on the structure of mate exchanges among members of the groups.

**Figure 9:**
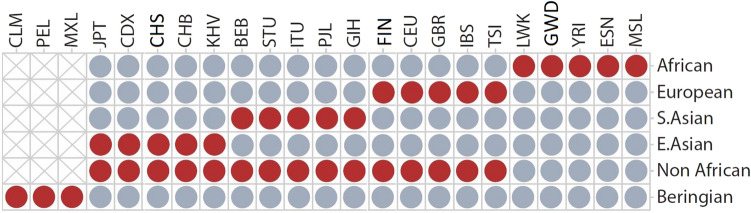
Search comparisons for GSPs. The strategy for a particular group identified alleles in all samples with red circles and absent or rare in all samples with blue circles. Samples with gray exes were left out of a particular comparison. The procedure was slightly modified for Indigenous American GSPs (see text). The populations and abbreviations are as follows: Colombian (CLM), Peruvian (PEL), Mexican Ancestry (MXL), Japanese (JPT), Chinese Dai (CDX), Chinese in South China (CHS), Chinese in Bejing (CHB), Kinh in Ho Chi Minh City, Vietnam (KHV), Bengali in Bangladesh (BEB), Sri Lankan Tamil in the UK (STU), Indian Telugu in the U.K. (ITU), Punjabi in Lahore, Pakistan (PJL), Gujarati Indians in Houston, Texas (GIH), Finn (FIN), Ceph European (CEU), British (GBR), Iberian (IBS), Toscani (TSI), Luhya in Webuye, Kenya (LWK), Gambian (GWD), Yoruba in Ibadan, Nigeria (YRI), Esanin (ESN), Mende in Sierra Leone (MSL).

The occurrence of GSPs depends on how groups of populations are defined. In this study, we consider the following groups determined by geographic locations, and descent groups formed by the well-documented out-of Africa migration that took place approximately 60,000 years before present^11,24,38^. To search for African, European, South Asian, East Asians, and non-African GSPs we made the comparisons illustrated in Fig. 9. Notably, we have not used the TGPP3 populations that reside in the Americas for these comparisons. This omission is necessary because the American populations likely harbor GSPs from throughout the world owing to formation by admixture in the Colonial era.

To identify Indigenous American GSPs it was necessary to control for non-indigenous American admixture. Thus, in the Mexican American sample, the 30% GSP threshold was transformed according to a 47% Native American ancestry component ($p = 0.30*0.47 = 0.14$) to yield a new GSP threshold of 14%. For the Peruvian sample, the adjusted threshold was $p = 0.23$, and for the Columbian sample the adjusted threshold was $p = 0.08$. An allele was considered a Beringian GSP if it met the modified criterion in all three American populations.

### Coalescent simulations

We used coalescent simulations to verify that the observed pattern of derived GSP alleles is consistent with the loss of variation from which the serial founder effects model was inferred^24,32^. Demographic parameters for these simulations were estimated by fitting a tree to data from the CEPH-HGDP short tandem repeat (STR) data set^39^.

We began by choosing a subset of 27 populations from the CEPH-HGDP dataset: San and Kxoe from South Africa; Mandenka, Brong, Igala, Yoruba, and Luhya from Central Africa; Russian, Tuscan, Orcadian, Basque, and French from Western Europe; Punjabi, Tamil, Bengali, Gujarati, and Telugu from South Asia; Cambodian, Dai, Han, North Chinese Han, and Japanese from East and South-East Asia; and Pima, Maya, Mixtec, Embera, and Cabecar who are Native Americans. The San and Kxoe served to root the tree for the remaining 25 populations^32,40^. The non-South African populations from Europe and Asia were chosen to match the populations in the thousand genomes project data as closely as possible. Native American populations were included to estimate demographic parameters for the Beringian Isolation period.

Next we used 619 microsatellite loci^39^ to compute Nei’s minimum genetic distances between all pairs of populations^41^. We re-scaled these distances by multiplying by 2. Using the re-scaled genetic distances, we built a neighbor-joining tree for the 27 populations. We rooted the neighbor-joining topology on the branch between the San-Kxoe and the remaining African and non-African populations, and then fitted branch lengths using the maximum-likelihood method proposed by Cavall-Sforza and Piazza^42–44^. The branch-lengths on the tree constructed in this manner measure the increase in gene identity (homozygosity) accrued between each pair of nodes moving from the root to the extant populations. The nodes on this tree were assigned chronological dates using estimates of the times at which modern humans inhabited the various regions of the globe. These dates were inferred from archaeological sources and independent genetic data^2,45,46^. We estimated effective population sizes for each branch of the tree by iteratively solving for the population size that would reproduce the genetic distance on that branch while allowing step-wise mutations to occur at a rate of $\mu = 10^{-4}$ per locus per generation . The fitted tree, branch points, node dates (in generations) and effective population sizes are provided as Supplementary Figure S2.

With the chronological dates and effective population size estimates obtained as outlined above, we simulated single-copy DNA sequences using an infinite sites mutation model and mutation rate of $\mu = 1.2 \times 10^{-8}$ per base pair per generation. The simulations projected DNA sequences in existing populations backwards in time through their history of changes in effective size at population splits. We performed these simulations using an original program that implements the algorithm of Hudson 1990^47^. The times of each mutation in the simulated coalescent histories were recorded, as were the frequencies of the mutant (derived) allele in the contemporary population. From this simulated data, we constructed the probability density for the age of a high frequency derived allele found in a population that inhabited a specific geographic region of the world. Thus, we were able to assess the probability that a derived allele arose, on a branch, at a time, that would render it exclusive to a particular set of populations or geographic region.

### Genome architecture and functional annotation

For comparative purposes, we constructed a random sample of 20,424 SNPs selected from across the genome. The allele frequency distribution and proportion of SNPs per chromosome in the random sample were matched to the set of Beringian GSPs that we discovered (See Results).

We searched for spatial clustering within the Beringian GSPs and random SNPs by tabulating the median distance from each SNP to its nearest neighbor within a window size of +/−10 SNPs.

We used the ANNOVAR annotation suite^48^ (https://rdocumentation.org/packages/annovarR/versions/1.0.0)bto categorize each variant from the Beringian GSPs and Random SNP Sets according to a variety of genomic properties. All SNPs were annotated as either intergenic, non-coding RNA (ncRNA), or genic (including introns, exons, UTRs). Exonic variants were further annotated to reflect synonymous and non-synonymous substitutions. We further annotated variants from the Beringian and Random SNP sets with known gene associations as reported by NCBI’s gene database. The gene associations included intergenic SNPs that fell within known regulatory regions of specific genes. Genes impacted by Beringian GSPs and Random SNPs were then grouped according to similar functional properties defined by both the Kyoto Encyclopedia of Genes and Genomes(KEGG) categories^49^ and gene ontology categories (GO) using the Enrichr https://cran.r-project.org/web/packages/enrichR/vignettes/enrichR.html and Panther version 16.0 (http://www.pantherdb.org/pathway/) software packages, respectively^33,50^.

### Detection of natural selection

To measure purifying selection, we calculated the $K_a/K_s$ ratio for genes with exonic GSPs following the method of Li et al.^51^ and compared it to the same measure on a random set of SNPs. Genic SNPs in both the Beringian and random sets were annotated as synonymous or non-synonymous substitutions using the ANNOVAR suite. To calculate $K_a$ (ratio of non-synonymous substitutions per non-synonymous site), we divided the total number of non-synonymous substitutions by the number of non-synonymous nucleotide sites for each gene with an exonic variant. Similarly, $K_s$ (ratio of synonymous substitutions per synonymous sites) was calculated as the ratio of synonymous substitutions to synonymous sites in the same gene set. Numbers of synonymous and non-synonymous sites were calculated as the weighted sum of probabilities that each site could experience a non-synonymous or a synonymous change. Finally, combining the data for all GSPs in exons, we calculated the ratio $\omega = K_a/K_s$ to determine whether the amino acid substitutions resulting from GSPs departed from the neutral expectation for neutral evolution $\omega = 1.0$^51^. To determine whether the GSPs displayed an atypical pattern of natural selection in comparison to SNPs chosen from the genome at random, we applied the above steps to the set of 20,424 random SNPs. $K_a/K_s$ ratios were computed in R 4.0.2 using the *seqinr* package version 4.2-5^52^ (http://seqinr.r-forge.r-project.org/) on gene sequences downloaded from the NCBI database.

To identify signals of positive selection, we calculated integrated haplotype homozygosity scores (*iHS*)^53^ using the *rehh* package in R^54^. We calculated an $|iHS |$ for each GSP, in each of the three American populations (MXL, CLM, PEL). According to standard practice, an $|iHS |$ score greater than 2.5 standard deviations from the mean is considered a candidate for positive selection; positive scores indicate selection favoring the ancestral allele, whereas negative scores indicate selection favoring the derived allele. The iHS statistic is useful for detecting selective sweeps that have not reached fixation and allows for prioritizing candidate SNPs, or genomic regions, but it does not a provide formal tests of significance.

In order to link natural selection and phenotypic targets for adaptation, we examine Beringian GSPs with high |*iHS*| scores three ways. First we present GSPs with the most extreme outlier |*iHS*| values. Next we identify which genes contain the greatest number of GSPs within the top 5% of iHS scores. Finally, to characterize biological systems and pathways affected by selected GSPs, we compiled a list of genes with Beringian GSPs within the top 5% of iHS scores and used the Enrichr tool^50^ to assess gene set enrichment in pathways described by the Kyoto Encyclopedia of Genes and Genomes (KEGG)^55^. Results from this analysis were rank ordered using the Enrichr combined score metric, which captures a measure of deviation between each category’s observed rank and the expected rank for that category by chance.

## Supplementary Information

Supplementary Information.

## References

[1] Mulligan CJ, Hunley K, Cole S, Long JC (2004). Population genetics, history, and health patterns in Native Americans. Annu. Rev. Genomics Hum. Genet..

[2] Kitchen A, Miyamoto MM, Mulligan CJ (2008). A three-stage colonization model for the peopling of the Americas. PLoS ONE.

[3] Reich D (2012). Reconstructing Native American population history. Nature.

[4] Tamm E (2007). Beringian standstill and spread of Native American founders. PLoS ONE.

[5] Pitulko VV (2004). The Yana RHS site: humans in the Arctic before the last glacial maximum. Science.

[6] Meiri M (2014). Faunal record identifies Bering isthmus conditions as constraint to end-Pleistocene migration to the New World. Proc. R. Soc. B Biol. Sci..

[7] Meiri WB (2012). Ecological studies of the UVB-vitamin D-cancer hypothesis. Anticancer Res..

[8] Fox-Dobbs K, Leonard JA, Koch PL (2008). Pleistocene megafauna from eastern Beringia: Paleoecological and paleoenvironmental interpretations of stable carbon and nitrogen isotope and radiocarbon records. Palaeogeogr. Palaeoclimatol. Palaeoecol..

[9] Hoffecker JF, Elias SA, O’Rourke DH, Scott GR, Bigelow NH (2016). Beringia and the global dispersal of modern humans: Beringia and the Global Dispersal of Modern Humans. Evolut. Anthropol. Issues News Rev..

[10] Mulligan CJ, Kitchen A, Miyamoto MM (2008). Updated three-stage model for the peopling of the Americas. PLoS ONE.

[11] Nielsen R (2017). Tracing the peopling of the world through genomics. Nature.

[12] Choin J (2021). Genomic insights into population history and biological adaptation in Oceania. Nature.

[13] Bergström A, Stringer C, Hajdinjak M, Scerri EML, Skoglund P (2021). Origins of modern human ancestry. Nature.

[14] Sikora M (2019). The population history of northeastern Siberia since the Pleistocene. Nature.

[15] Hoffecker JF, Powers WR, Goebel T (1993). The colonization of Beringia and the peopling of the New World. Science.

[16] Hoffecker JF, Elias SA, O’Rourke DH (2014). Out of Beringia?. Science.

[17] Santos FR (1999). The central Siberian origin for native American Y chromosomes. Am. J. Human Genet..

[18] Wilson JF (2001). Population genetic structure of variable drug response. Nat. Genet..

[19] Schurr TG (2004). The peopling of the new world: Perspectives from molecular anthropology. Annu. Rev. Anthropol..

[20] Bonatto SL, Salzano FM (1997). A single and early migration for the peopling of the Americas supported by mitochondrial DNA sequence data. Proc. Natl. Acad. Sci..

[21] Silva WA (2002). Mitochondrial genome diversity of native Americans supports a single early entry of founder populations into America. Am. J. Human Genet..

[22] Nei Masatoshi, Maruyama Takeo, Chakraborty Ranajit (1975). The Bottleneck effect and genetic variability in populations. Evolution.

[23] Kimura M (1955). Random genetic drift in multi-allelic locus. Evolution.

[24] Ramachandran S (2005). Support from the relationship of genetic and geographic distance in human populations for a serial founder effect originating in Africa. Proc. Natl. Acad. Sci. U.S.A..

[25] Lohmueller KE (2008). Proportionally more deleterious genetic variation in European than in African populations. Nature.

[26] Lohmueller KE (2014). The distribution of deleterious genetic variation in human populations. Curr. Opin. Genet. Dev..

[27] Schroeder K (2007). A private allele ubiquitous in the Americas. Biol. Let..

[28] Amorim GCE (2017). Genetic signature of natural selection in first Americans. Proc. Natl. Acad. Sci..

[29] Hlusko LJ (2018). Environmental selection during the last ice age on the mother-to-infant transmission of vitamin D and fatty acids through breast milk. Proc. Natl. Acad. Sci..

[30] Gravel S (2011). Demographic history and rare allele sharing among human populations. Proc. Natl. Acad. Sci..

[31] Moreno-Estrada A (2013). Reconstructing the population genetic history of the Caribbean. PLoS Genet..

[32] Li JZ (2008). Worldwide human relationships inferred from genome-wide patterns of variation. Science.

[33] Thomas PD (2003). PANTHER: a library of protein families and subfamilies indexed by function. Genome Res..

[34] Stelzer G (2016). The GeneCards suite: from gene data mining to disease genome sequence analyses. Curr. Protoc. Bioinformatics.

[35] Jablonski NG, Chaplin G (2010). Human skin pigmentation as an adaptation to UV radiation. Proc. Natl. Acad. Sci..

[36] The 1000 Genomes Project Consortium. A global reference for human genetic variation. *Nature* **526**, 68–74 (2015) 10.1038/nature15393 PMC4750478 26432245

[37] Martin AR (2017). Human demographic history impacts genetic risk prediction across diverse populations. Am. J. Human Genet..

[38] Coop G (2009). The role of geography in human adaptation. PLoS Genet..

[39] Pemberton TJ, DeGiorgio M, Rosenberg NA (2013). Population structure in a comprehensive genomic data set on human microsatellite variation. G3: Genes|Genomes|Genetics.

[40] Mallick S (2016). The Simons Genome Diversity Project: 300 genomes from 142 diverse populations. Nature.

[41] Nei M (1987). Molecular evolutionary genetics.

[42] Cavalli-Sforza LL, Piazza A (1975). Analysis of evolution: Evolutionary rates, independence and treeness. Theor. Popul. Biol..

[43] Long JC, Kittles RA (2003). Human genetic diversity and the nonexistence of biological races. Hum. Biol..

[44] Urbanek M, Goldman D, Long JC (1996). The apportionment of dinucleotide repeat diversity in Native Americans and Europeans: A new approach to measuring gene identity reveals asymmetric patterns of divergence. Mol. Biol. Evol..

[45] Bae CJ, Douka K, Petraglia MD (2017). On the origin of modern humans: Asian perspectives. Science.

[46] Graf KE, Buvit I (2017). Human dispersal from Siberia to Beringia: assessing a Beringian Standstill in light of the archaeological evidence. Curr. Anthropol..

[47] Hudson RR (1991). Gene genealogies and the coalescent process. Oxford Surv. Evolut. Biol..

[48] Wang K, Li M, Hakonarson H (2010). ANNOVAR: functional annotation of genetic variants from high-throughput sequencing data. Nucleic Acids Res..

[49] Kanehisa M (2019). Toward understanding the origin and evolution of cellular organisms. Protein Sci. Publ. Protein Soc..

[50] Chen EY (2013). Enrichr: interactive and collaborative HTML5 gene list enrichment analysis tool. BMC Bioinformatics.

[51] Li W-H (1993). Unbiased estimation of the rates of synonymous and nonsynonymous substitution. J. Mol. Evol..

[52] Charif D, Lobry JR, Bastolla U, Porto M, Roman HE, Vendruscolo M (2007). SeqinR 1.0-2: A contributed package to the r project for statistical computing devoted to biological sequences retrieval and analysis. Structural approaches to sequence evolution: molecules, networks, populations.

[53] Voight BF, Kudaravalli S, Wen X, Pritchard JK (2006). A map of recent positive selection in the human genome. PLoS Biol..

[54] Gautier M, Vitalis R (2012). Rehh: an R package to detect footprints of selection in genome-wide SNP data from haplotype structure. Bioinformatics.

[55] Kanehisa M, Goto S (2000). KEGG: Kyoto encyclopedia of genes and genomes. Nucleic Acids Res..

