# Supplementary material for: Novel alleles gained during the Beringian isolation period
Source: Sci Rep. 2022 Mar 11;12:4289. doi: 10.1038/s41598-022-08212-1 (PMC8917172; doi:10.1038/s41598-022-08212-1)
Supplement: Supplementary file 1 — Supplementary Information. [file 41598_2022_8212_MOESM1_ESM.pdf]

Supplementary Information for *Novel Alleles Gained During the Beringian Isolation Period.*

Sara D. Niedbalski and Jeffrey C. Long.

Supplementary Table S1

Thousand Genomes Project Phase III Populations used in this project.

Label	N	Name and Location	Region of Origin
MSL	85	Mende in Sierra Leone	W. Africa
ESN	99	Esanin Nigeria	W. Africa
YRI	108	Yoruba in Ibadan, Nigeria	W. Africa
GWD	113	Gambian in Western Division	W. Africa
LWK	99	Luhya in Webuye, Kenya	E. Africa
TSI	107	Toscani in Italia	S. Europe
IBS	107	Iberian Populations in Spain	S. Europe
GBR	91	British from England and Scotland	N. Europe
CEU	99	Ceph European	European Immigrants
FIN	99	Finnish in Finland	N. Europe
GIH	103	Gujarati Indians in Houston, Texas	S. Asia
PJL	96	Punjabi in Lahore, Pakistan	S. Asia
ITU	102	Indian Telugu in the U.K.	S. Asia
STU	102	Sri Lankan Tamil in the UK	S. Asia
BEB	86	Bengali in Bangladesh	S. Asia
KHV	99	Kinh in Ho Chi Minh City, Vietnam	S.E. Asia
CHB	103	Chinese in Bejing	E. Asia
CHS	105	Han Chinese South	E. Asia
CDX	93	Chinese Dai in Xishuangbanna, China	E. Asia
JPT	104	Japanese in Tokyo, Japan	E. Asia
MXL	64	Mexican Ancestry in Los Angeles	Immigrants from Mexico
CLM	94	Colombian in Medellin, Colombia	S. America
PEL	85	Peruvian in Lima, Peru	S. America

Suppementary Figure S1

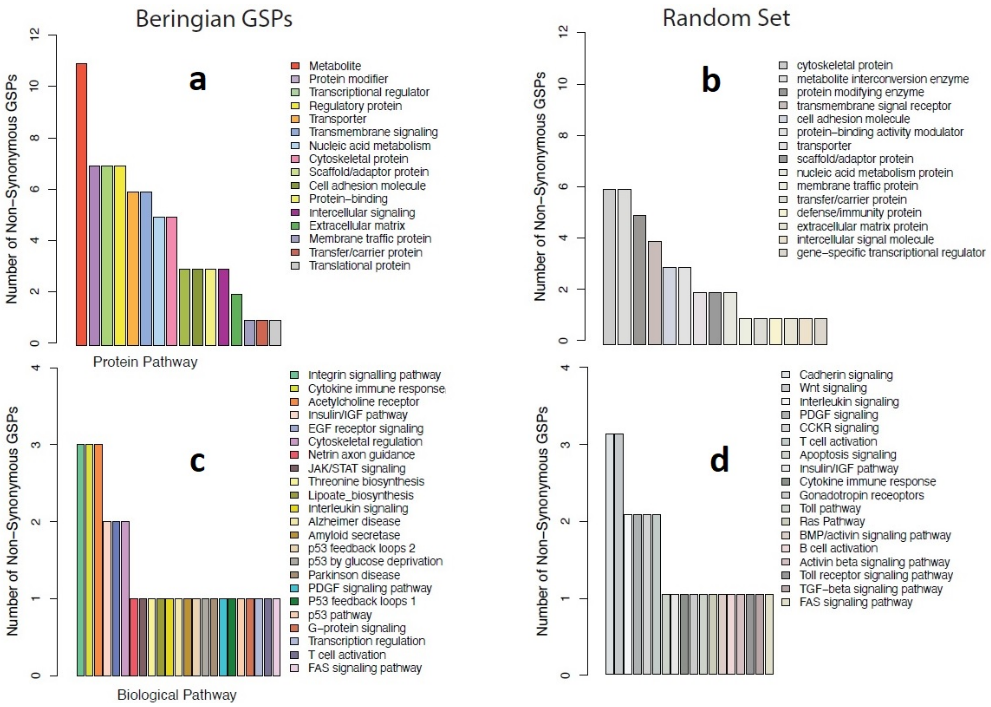

Panther annotations for non-synonymous GSPs compared to Panther annotations for a random sample of 20,424 matched SNPs chosen across the genome (See Methods). Panel **a** depicts the protein classes affected by non-synonymous variation unique to Beringia. Panel **b** depicts the protein classes affected by the randomly chosen SNPs. Panel **c** annotates the Beringian protein coding changes according to which biological pathway they are implicated in. Panel **d** annotates the random coding SNPs according to which biological pathway they are implicated in.

Supplementary Figure S2

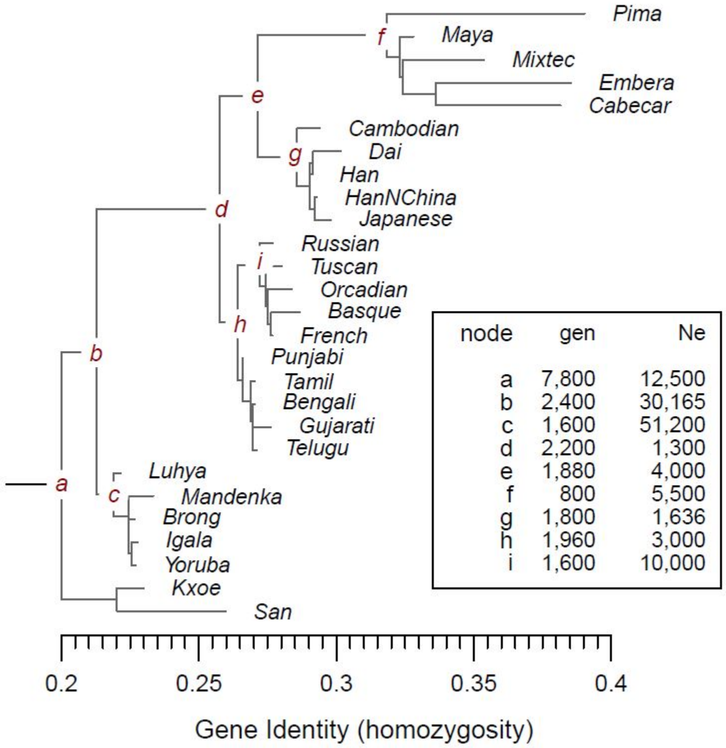

Tree of relationships inferred for 27 human populations. Dates are given in generations (25 years) before present for the principal nodes of the tree. N_e listed for each node denotes effective population size along the branch leading into the node.
